# Geriatric Oncology in the Instagram Era: Feasibility and Acceptability Randomised Controlled Trial on Adopting PhotoVoice to Enable Empowerment, Patient-Centred Care, and Shared Decision Making—Study Protocol

**DOI:** 10.3390/mps6040068

**Published:** 2023-07-26

**Authors:** Christopher Steer, Tshepo Rasekaba, Kylie Owen, Darren Jayasuriya, Mira Kapur, Kim Young, Nicole Webb, Irene Blackberry

**Affiliations:** 1 John Richards Centre for Rural Ageing Research, La Trobe Rural Health School, La Trobe University, Albury-Wodonga, VIC 3690, Australia; christopher.steer@bordermedonc.com.au (C.S.); sw2kimy@gmail.com (K.Y.); i.blackberry@latrobe.edu.au (I.B.); 2 Border Medical Oncology and Haematology, Albury-Wodonga, NSW 2640, Australia; nicole.webb@awh.org.au; 3 School of Clinical Medicine, Rural Clinical Campus, University of New South Wales, Albury, NSW 2640, Australia; d.jayasuriya@student.unsw.edu.au (D.J.); m.kapur@unsw.edu.au (M.K.); 4 Department of Community and Clinical Allied Health, La Trobe Rural Health School, La Trobe University, Albury-Wodonga, VIC 3690, Australia; kylie.owen@nhw.org.au; 5 Care Economy Research Institute, La Trobe University, Albury-Wodonga, VIC 3690, Australia

**Keywords:** geriatric assessment, PhotoVoice, enhanced supportive care, geriatric oncology, patient-centred care

## Abstract

Geriatric assessment (GA) is fundamental to optimising cancer care in older adults, yet implementing comprehensive GA tools in real-world clinical settings remains a challenge. This study aims to assess the feasibility and acceptability of integrating information from patient-derived photographs (PhotoVoice) into enhanced supportive care (ESC) for older adults with cancer. A feasibility randomised controlled trial will be conducted at a regional cancer care centre in Australia. Participants aged 70 and above will be randomised into two groups: PhotoVoice plus ESC or usual care (ESC) alone. In the PhotoVoice group, participants will provide four photographs for deduction of representations of different aspects of their lives using photo-elicitation techniques. ESC will be conducted for both groups, incorporating PhotoVoice analysis in the intervention group. PhotoVoice may improve patient-centred care outcomes, including enhanced communication, shared decision making, and identification of patient priorities and barriers. Findings will provide insights into implementing PhotoVoice in geriatric assessment and guide future trials in cancer among older adults.

## 1. Background

Age is a significant risk factor for cancer, and the ageing process plays a crucial role in cancer progression and care. Older individuals aged 65 years and above account for a substantial proportion of cancer diagnoses and deaths [1,2]. Consequently, frail older adults are more susceptible to complications, treatment toxicity, increased hospitalisation, and mortality [3,4].

The ageing process is complex and heterogeneous, necessitating treatment decisions that go beyond chronological age and consider various biopsychosocial factors. These factors include comorbidities, geriatric syndromes, functional status, nutritional health, and socioeconomic characteristics [5,6,7]. Therefore, it is crucial to incorporate geriatric assessment (GA)-guided interventions, which consider frailty, comorbidity, and psychosocial domains, into the cancer care of older adults [5,8].

To improve the quality of care and address age-related concerns, multidimensional assessment of older adults with cancer has been shown to be beneficial. This approach enables personalised, patient-centred supportive care [1,2]. However, the implementation of such assessments is often suboptimal, particularly in regional and rural centres that encounter challenges such as a shortage of skilled healthcare professionals. This issue is particularly important when considering the critical role of a multidisciplinary team (MDT) in providing holistic cancer care. The integration of all professionals involved in the treatment decision-making process for older people with cancer enhances patient-centred supportive care during diagnosis, treatment, and follow-up periods, as well as palliative and end-of-life care management, and it is appreciated by most patients [5]. The objective of the MDT is to provide comprehensive, high-quality care that is congruent with the patient’s goals [9] identified through GA. Evidence from randomised control trials suggests that geriatric assessment guided interventions improve quality of life, decrease treatment-related toxicity, and reduce healthcare utilisation in older adults with cancer [10,11].

In geriatric oncology, MDT is defined as the collaboration between professionals from different specialised fields of practice who work together with the goal of enhancing patient care and treatment efficacy whilst minimising toxicity [5]. Geriatric oncology MDTs are usually made up of medical oncologists, geriatricians, oncology nurses, social workers, pharmacists, palliative care specialists, primary care physicians, nutritionists/dietitians, occupational therapists, physical therapists [3,9], psychologists, speech pathologists, nursing teams [12], and pastoral care [13].

Best practice guidelines for cancer care in older adults emphasise the importance of GA as a foundation for shared treatment decision making. The International Society of Geriatric Oncology (SIOG) recommend implementing GA for shared treatment decision-making in older adults with cancer [14,15]. The National Comprehensive Cancer Network (NCCN) guidelines highlighted 28 risk factors, including geriatric syndromes, comorbidities, and socioeconomic issues, that impact treatment outcomes and require assessment before recommending anticancer therapy [16]. The foundation of geriatric medicine lies in the utilisation of multidimensional GA, which is then followed by GA-informed intervention recommendations [17,18]. These recommendations are incorporated into patient-centred care plans, accompanied by ongoing follow-up.

The integration of geriatricians into MDTs and the routine use of GA in oncology practice have enhanced the care of older cancer patients [12,18]. Older adults who perform poorly on the GA have adverse outcomes after undergoing surgery including increased risk of post-operative mortality [7]. GA has been shown to have multiple benefits, including predicting treatment-related toxicities; assessing frailty accurately; guiding shared decision making; and informing non-oncologic interventions such as social support, polypharmacy management, nutrition interventions, physiotherapy, and psychological care [3,16]. These non-oncological interventions have been shown to enhance patient adherence, treatment tolerance, and improve quality of life [5].

Despite growing recognition of the importance of GA in guiding geriatric oncological treatment decisions, implementation remains limited. Only 36% of surgical oncologists collaborate with geriatricians, and 48% consider GA essential for decision making [19]. Time constraints and performance targets driven practice and MDTs that focus primarily on cancer pathology and treatment may neglect essential patient-centred information, leading to overtreatment and poor outcomes [3,20]. As such, there is a need to explore innovative methods to streamline assessment information gathering. 

PhotoVoice is a qualitative research method that utilises photography and storytelling [21,22]. In cancer care, PhotoVoice could be used to explore the patient’s experience and perception of cancer. It may enhance clinicians’ understanding of older patients’ lived experiences, empower patients, and increase self-awareness [21]. By capturing the strengths and challenges of an older adult’s environment, PhotoVoice may optimise person-centred care [23]. It enables patients to take the lead in discussions, providing richer descriptions and addressing important topics [22] that may be missed during busy routine practice. Moreover, PhotoVoice promotes patients’ awareness of the factors influencing their health and treatment decisions [24]. In conventional photovoice, participants typically receive a camera to capture photos for later discussion [23]. In our proposed study, participants will provide photographs they select from their existing collection.

Our proposed study will investigate the utility of integrating PhotoVoice discussion of patient-supplied photographs into GA-guided ESC and examine its potential to contribute to patient-centred care. Enhanced Supportive Care refers to a comprehensive approach that engenders addressing the physical, emotional, social, and practical needs of patients with cancer [25,26]. It involves providing additional interventions, services, and resources to optimise the overall well-being and quality of life of patients throughout their cancer journey [25].

Implementation of PhotoVoice in our study is undergirded by the Bronfenbrenner model [27]. The model is based on the theory that understanding individuals and their behaviours is underpinned by the reciprocal interaction between individuals and their environment. Its domains include the individual, microsystem (immediate relationships), mesosystem (connections between microsystems), exosystem (indirect influences), and macrosystem (broader cultural factors), representing the multidimensional nature of development and its interconnected influences [27].

The study objective is to investigate the feasibility and acceptability of implementing PhotoVoice in addition to usual care in a regional cancer centre. Usual care in this context is defined as ESC that incorporates geriatric assessment. Given the early stages of ESC, a secondary aim is to undertake a quality improvement exercise to examine aspects of ESC adoption using a medical records audit. The primary and secondary research questions are, respectively, (i) what is the feasibility, acceptability, and utility of PhotoVoice to enable empowerment, patient-centred care, and shared decision making for older adults diagnosed with a cancer in a regional cancer centre? and (ii) using a medical records audit, what is the level of uptake of supportive (non-medical) care practice as demonstrated by a range of tools as proxies for supportive care including use of the distress thermometer (DT), electronic rapid fitness assessment (eRFA) and referrals to supportive care services before and during the implementation of PhotoVoice?

## 2. Methods

### 2.1. Study Design

**Figure 1:**
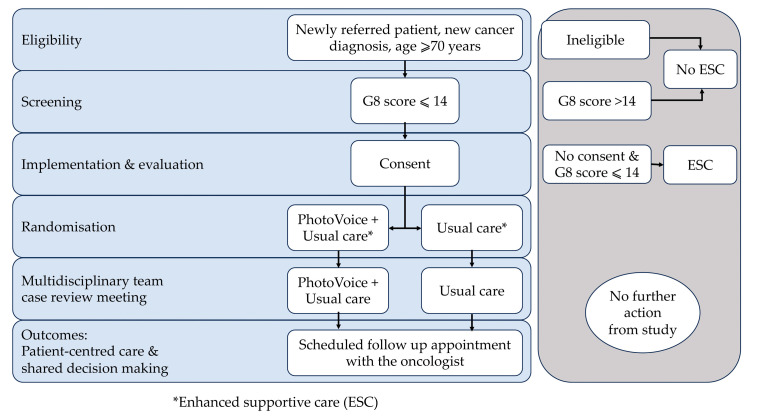
Summary of study design and participant flow.

The study will be a feasibility and acceptability randomised controlled trial comparing PhotoVoice-augmented ESC with usual care in a regional geriatric oncology service (Figure 1). The RCT will be supplemented with a pre and post clinical quality improvement audit to evaluate GA use in the cancer centre.

### 2.2. Population and Setting

This study will involve older adults aged 70 years and above who have been diagnosed with a new cancer and referred to Border Medical Oncology and Haematology (BMOH) at the Albury Wodonga Regional Cancer Centre (AWRCC). This specialised cancer centre is located on the border between New South Wales and Victoria in regional Australia. BMOH provides care to a population of patients with cancer, including an annual intake of nearly 800 individuals aged over 69 years. The most frequently observed tumour types at BMOH include breast, prostate, colorectal, lung cancer, and melanoma.

## 3. Procedure

### 3.1. Screening

Prior to obtaining consent and randomisation, potential participants will undergo routine screening using the simple but sensitive G8 tool designed for assessing frailty among elderly patients who have been diagnosed with cancer [17]. The G8 is a validated geriatric screening tool consisting of eight items, which is based on the Mini Nutritional Assessment [28]. It has been validated and used in older patients with cancer [17,29]. Patients who score ≤14 on the G8 are considered vulnerable and therefore more likely to benefit from a comprehensive geriatric assessment [6], thus being eligible for the study. Eligible patients will be randomised to PhotoVoice plus usual care or usual care alone.

### 3.2. Inclusion and Exclusion Criteria

Prospective participants aged ≥70 years, with a diagnosis of cancer and newly referred for treatment at the AWRCC, scoring ≤14 on the self-reported G8 tool, and providing informed consent (including via an authorised proxy) will be included. Patients receiving treatment as an in-patient in the AWRCC will be excluded.

### 3.3. Randomisation

Participants will be randomly assigned to either PhotoVoice and ESC (Intervention) group or ESC only (Control) using a 1:1 computer-generated randomisation schedule created by an independent researcher. Allocation numbers will be placed in sealed opaque envelopes and drawn sequentially for each eligible participant.

### 3.4. Intervention (PhotoVoice Optimised ESC)

The intervention follows the usual care process described below, with the addition of PhotoVoice as a strategy to enhance the collection of patient-centred information. Participants will be asked to provide four photographs based on the following themes informed by the Bronfenbrenner model [27]:

- A photograph depicting an aspect of their identity, career, workplace, or life role, emphasising their personal journey.
- A photograph representing something important to the patient, such as a pet or family member.
- A photograph showcasing the patient’s home environment.
- A photograph illustrating their means of transportation to appointments.

These photographs will be discussed using established PhotoVoice photo-elicitation techniques based on the Modified SHOWeD questions [30]. The questions will guide the discussion, exploring the meaning and relevance of the images to the patient’s life and care. They may focus on identifying strengths, problems, and potential actions for patient-centred care.

The MDT will review the findings from the assessments and incorporate the analysis of the PhotoVoice images. This combined information will inform the care decision-making discussion and development of a management plan that aligns with the patient’s priorities. Depending on the identified deficits, impairments, and the patient’s preferences and strengths as gleaned from the PhotoVoice and MDT discussions, referrals may be made to services such as allied health, social supports, and assistive equipment services, etc.

### 3.5. Usual Care (ESC)

The care pathway includes screening and assessment tools including the G8 [17], the mood disorders screening DT [31], eRFA [32], body mass index (BMI) measurement, the Timed Up and Go (TUG) [33], and the Mini-COG for cognitive impairment screening [34]. The eRFA is a multidimensional electronic geriatric assessment questionnaire that takes around 15 min to complete on a tablet or computer [32]. The TUG test is a widely used tool for the assessment of a patient’s mobility, balance, and transfer abilities, thus assessing limitations in their functional activities [33]. At the initial consultation, patients undergo these assessments with the supervision of a cancer care nurse coordinator (G8 and DT). Patients scoring ≤14 on the G8 complete the eRFA on a tablet device, in addition to the TUG and Mini-COG.

The results are evaluated by the geriatric oncology MDT during their weekly meeting, which consists of various healthcare professionals. Based on this evaluation, personalised supportive care recommendations are made. The treating oncologist utilises the MDT’s evaluation report to inform treatment and care decisions.

### 3.6. Recruitment

After obtaining ethical approval, all patients presenting for their first appointment with any of the medical oncologists or haematologists during the period from February 2021 to January 2022 with a G8 score ≤14 would be offered enrolment in the study. The attending medical oncologist or haematologist would provide the patient with a participant information and consent document. Patients expressing interest in the study would be referred to the research team member (for a detailed study briefing), followed by obtaining informed consent. Prospective participants requiring more time to decide on participation would be given up to two business days to consider before following up if they had not responded.

All members of the MDT will receive a study briefing and invitation to participate during their weekly team meeting. They will be asked to complete a clinician survey (Appendix A), and consent will be implied by returning a completed survey.

### 3.7. Data Collection and Analysis

Demographic and characteristic data, including age, gender, cancer diagnosis, and comorbidities, will be collected from participants who provide a signed consent. Participant characteristics will be summarised using descriptive statistics, such as means and standard deviations for continuous variables, median and interquartile ranges for ordinal variables, and percentages for categorical variables.

Once the photographs have been collected within the next 1–2 scheduled appointments with the oncologist, a researcher will conduct a telephone or Zoom interview (Appendix B) with the patient, using PhotoVoice photo elicitation techniques to explore their priorities, barriers, and supports. The audio recorded interview will be transcribed verbatim and followed by thematic analysis. Themes will be guided by and grouped into the Bronfenbrenner model domains [27] to understand the older adult diagnosed with cancer as an individual in and how they influence and are influenced their social environment. A summary case report presenting the photographs along with a brief interpretation of their implications and salient aspects will be generated for the MDT meeting timed to occur before scheduled oncology appointments 2–3. As authorised proxies will be permitted to provide consent for participation, they will also be allowed to assist or provide responses on behalf of participating patients.

For participants in the usual care group, data collection will include a management plan based on the patient’s priorities determined through the usual care process.

In addition, surveys will be administered to both participating patients (Appendix C) and clinicians involved in the study (Appendix A). The tools used for the surveys will include the Health Care Climate Questionnaire [10,35,36,37], Perceived Efficacy in Patient-Physician Interactions Scale [10,38], Control Preferences Scale [10,39], and Treatment Decision-Making Form [10].

The implementation and impact of ESC on patient communication regarding age-related concerns will be compared before and after the first visit. Patient-centred care and shared decision-making outcomes between the PhotoVoice plus ESC and ESC only will be compared. Follow-up outcome data collection will be timed to take place 4–8 weeks after the MDT meeting.

### Supplementary Sub-Study: Quality Improvement Audit of ESC Uptake

To evaluate the practice of ESC, a quality improvement exercise will be conducted to assess the utilisation of ESC before and during the proposed pilot feasibility RCT detailed above. This evaluation will employ a medical records audit methodology, which is a recognised approach for examining the delivery of clinical care and identifying areas for improvement [40]. Data will be collected using a purpose-designed checklist (Appendix D) to guide data extraction for any ESC aligned assessment and practice elements such as the DT, eRFA, and supportive care referrals, i.e., allied health and social support services.

The audit process will involve randomly selected patient records for each audit phase (before, during, and after) based on unique record numbers obtained from the reception staff. These record numbers will be assigned an Audit ID and then randomly selected using an Excel random number generator.

The sample size for the audit will depend on the specific phase being evaluated. For the baseline phase (pre-PhotoVoice optimised ESC), assuming N = 800 patient records for the calendar year 2020 and an estimated 10% ad hoc use of ESC before formal implementation (*p* = 0.1), a sample size of 118 records will be audited to achieve a 95% level of confidence (Z = 1.96) and a 5% margin of error (E = 0.05) and relative standard error (RSE) = 0.5. This is based on the desired proportion (10%) sample size formula n = (N × Z^2 × *p* × (1 − *p*))/[(N − 1) × E^2 + Z^2 × *p* × (1 − *p*)/(N × RSE^2)].

During the three months of PhotoVoice-optimised ESC as a structured implementation process, assuming 200 eligible records (a quarter of the annual throughput) and a 50% adoption of ESC, a sample size of 132 records will be randomly selected and audited to achieve a 95% level of confidence and a 5% margin of error and RSE = 0.5.

The audit data will be analysed using summary descriptive statistics, including means, standard deviations, proportions, and 95% confidence intervals. Comments from the audit checklist will be subjected to content and/or thematic analysis.

## 4. Expected Results

By capturing older adult patients’ perspectives, priorities, and barriers during geriatric assessment, we anticipate that PhotoVoice will provide valuable insights into the acceptability and benefits of this novel approach. Successful integration of PhotoVoice into the ESC framework has the potential to attract interest in the geriatric assessment space by empowering healthcare providers to develop tailored management plans that align precisely with the unique needs and preferences of older adults with cancer. This approach has the capacity to bridge communication gaps, improve shared decision making, and enhance the overall care experience for the older adult population undergoing treatment for cancer. By taking cognisance of the voices and perspectives of older patients, this study could promote better outcomes and improved quality of life. The integration of PhotoVoice into ESC signifies commitment to bridging communication gaps and fostering meaningful engagement between healthcare providers and older adults diagnosed with cancer. Expanding understanding of how PhotoVoice may enhance geriatric assessment, could promote personalised care that addresses the needs and concerns of older adults, ultimately improving their treatment experiences and overall well-being during their cancer care journey. Our findings will contribute to the growing body of evidence supporting the integration of patient-centred approaches in geriatric oncology.

Finally, identifying enablers and barriers during the implementation process may lead to refining the intervention process and optimise its effectiveness. The findings will guide future advancements, ultimately informing the design of large-scale implementation and effectiveness trials. This could pave the way for broader integration of the PhotoVoice approach into geriatric assessment across diverse healthcare settings.

## 5. Conclusions

This study aims to investigate the use of PhotoVoice discussion of patient-derived photographs, a novel methodology in the context of cancer care geriatric assessment and ESC in a regional cancer care centre. The integration of PhotoVoice into the ESC framework has the potential to attract interest that fosters the development of tailored management plans that align with the unique needs and preferences of older adults diagnosed with cancer. Beyond immediate implementation considerations, this study may contribute to the broader field of geriatric oncology. The findings could potentially contribute to enhancing patient-centred care and pave the way for further research in this field, particularly in a definitive implementation and effectiveness trial of PhotoVoice.

## Data Availability

Not applicable.

## Appendix A. Clinician Survey

1. Please tell us about yourself:
1.1. Discipline/Specialty:

Allied health
Medical (physician, GP)
Nursing
Non-clinical supportive service

1.2. Years in practice: _________________________________
1.3. Do you have experience in cancer care: Yes/No

If yes, how many years of experience? ________

2. What did you like about the ESC initiative? ___________________________________________________________________________________________________________________________________________________________________________________________________________________________________________________________________________________________________________________________
3. What did you dislike about the ESC initiative? ____________________________________________________________________________________________________________________________________________________________________________________________________________________________________________________________________________________________________________________________
4. In thinking about the multidisciplinary team meetings:
4.1. How many meetings did you attend Between February and May 2021 (NB. date will be adjusted depending on when meetings formally start)? ____________________
4.2. Please rate the MDT meetings on the following
4.2.1. Time of the meeting

Suitable
Unsuitable
Preferred a different time (specify): _____________________

Comments:
____________________________________________________________________________________________________________________________________________________________________________________________________________________________________________________________________________________________________________________________

4.2.2. Frequency of the meeting

Too long
Too short
Just right

4.2.3. Length of the meeting

Too long
Too short
Just right

4.2.4. In thinking about the meetings, you attended in general, what did you think of content and discussion?

too much information
too little information
just right

Comments:
__________________________________________________________________________________________________________________________________________________________________________________________________________________________________________________________________________________________________________________________

4.3. How useful did you find the ESC weekly MDT meetings when you were making your decision about providing personalised patient care and support?

Unhelpful
Fair
Good
Excellent

Comments:
____________________________________________________________________________________________________________________________________________________________________________________________________________________________________________________________________________________________________________________________

5. In thinking about PhotoVoice and the discussions based on reports generated from this exercise, how useful did you find the PhotoVoice reports when you were making your decision about providing personalised patient care and support?

Unhelpful
Fair
Good
Excellent

5.1. To what extent did PhotoVoice help, if at all, clarify understanding the potential strengths and challenges within the older person’s environment?

Unhelpful
Fair
Good
Excellent

5.2. PhotoVoice was intended to provide a deeper exploration and appreciation for the patient’s priorities and response to their treatment decisions. How helpful do you think PhotoVoice was in achieving this intent?

Unhelpful
Fair
Good
Excellent

6. What did you like about PhotoVoice?

____________________________________________________________________________________________________________________________________________________________________________________________________________________________________________________________________________________________________________________________

7. What did you dislike about PhotoVoice?

____________________________________________________________________________________________________________________________________________________________________________________________________________________________________________________________________________________________________________________________

8. Do you have any suggestions to improve ESC, including Photovoice or any other suggestions?

____________________________________________________________________________________________________________________________________________________________________________________________________________________________________________________________________________________________________________________________

## Appendix C. Patient Survey

Health Care Climate Questionnaire and Health Care Climate Questionnaire Modified for Aging-Related Concerns

**Satisfaction with Overall Communication about Overall Health**	**Strongly Agree**	**Agree**	**Neutral**	**Disagree**	**Strongly Disagree**
1. Your cancer doctor encouraged you to **ask questions**.					
2. Your cancer doctor was **willing to discuss** any topic of importance to you.					
3. Your cancer doctor **gave you information** you could understand.					
4. Your cancer doctor helped you to **feel comfortable** discussing what to expect in the future.					
5. You feel **understood** by your cancer doctor.					

**Satisfaction with Communication about Other Medical Issues and Aging Concerns**	**Strongly Agree**	**Agree**	**Neutral**	**Disagree**	**Strongly Disagree**
1. Your cancer doctor encouraged you to **ask questions** about your other medical issues in addition to the cancer and/or any health concerns that could be from aging.					
2. Your cancer doctor was **willing to discuss** your other medical issues in addition to the cancer and/or any health concerns that could be from aging.					
3. Your cancer doctor **gave you information** you could understand about your other medical issues in addition to the cancer and/or any health concerns that could be from aging.					
4. Your cancer doctor helped you to **feel** **comfortable** discussing how cancer treatment could affect your other medical issues in addition to the cancer and/or any health concerns that could be from aging.					
5. You feel your cancer doctor **understood your overall health**, including your other medical issues in addition to the cancer and/or any health concerns that could be from aging.					
6. I understand why my cancer doctor suggested my treatment plan because he/she **talked with me about my medical tests** and procedures and how it led to my current diagnosis.					
7. You feel your cancer doctor **understood you** as a person, including values and beliefs important to you.					

## Appendix D. Audit Checklist

Patient’s Age: ________
Gender: ☐Male ☐Female ☐Other
ATSI: ☐Yes ☐No
Diagnosis: _________________________________________________
Treatment regimen: _________________________________________

**Item**	**Yes/No**	**Comments**
G8	☐Yes ☐No	Score if yes:
DT	☐Yes ☐No	
eRFA	☐Yes ☐No	
TUG	☐Yes ☐No	
Mini-Cog	☐Yes ☐No	
Allied health referral(s)	☐Yes ☐No	
If yes for above, specify		
Other supportive care services	☐Yes ☐No	
If yes for above, specify		
